# Cutaneous T-cell lymphoma in erythrodermic cases may be suspected on the basis of scalp examination with dermoscopy

**DOI:** 10.1038/s41598-020-78233-1

**Published:** 2021-01-11

**Authors:** Adriana Rakowska, Magdalena Jasińska, Mariusz Sikora, Joanna Czuwara, Patrycja Gajda-Mróz, Olga Warszawik-Hendzel, Małgorzata Kwiatkowska, Anna Waśkiel-Burnat, Małgorzata Olszewska, Lidia Rudnicka

**Affiliations:** 1 Department of Dermatology, Medical University of Warsaw, Koszykowa 82a, 02-008 Warsaw, Poland; 2 Alfa-Lek Medical Center, Nowy Swiat 58, 00-363 Warsaw, Poland

**Keywords:** Cancer, Diseases, Cancer, Skin diseases

## Abstract

Erythrodermic variants of cutaneous T-cell lymphoma (CTLC) are one of the case of erythroderma. The aim of the study was to assess the value of scalp dermoscopy in differentiation between erythrodermic CTCL, psoriasis, and atopic dermatitis. A total of 76 patients were included into the study **(**16 patients with erythrodermic CTCL, 20 patients with psoriatic erythroderma, 20 with erythrodermic atopic dermatitis, and 20 healthy volunteers). The most common trichoscopic features of erythrodermic CTCL were: numerous *pili torti*, numerous broken hairs, white thick interfollicular bands, and patchy hyperpigmentation of the background. They were observed in 81% (13/16), 75% (12/16), 56% (9/16), and 37.5% (6/16) of patients with CTCL, respectively (p < 0.001). Other specific features of erythrodermic CTCL were 8-shaped hairs (19%; 3/16) and visible anagen bulbs (12.5%; 2/16) (p < 0.05 and p = 0.052, respectively). The most common vascular pattern of erythrodermic CTCL was perifollicular arrangement of glomerular (50%; 8/16; p < 0.001) or linear vessels (31%; 5/16; p < 0.05). Follicular spicules-like scaling was pathognomonic for erythrodermic CTCL (12%, 2/16) although its presence did not reach statistical significance (p = 0.052). In conclusion, the characteristic trichoscopic findings of erythrodermic CTCL are numerous *pili torti*, eight-shaped hairs, thick white interfollicular bands, color heterogeneity of the background and perifollicular arrangement of vessels.

## Introduction

The diagnosis of erythroderma at any age is challenging if there is no previous history of psoriasis or atopic dermatitis. Erythrodermic variants of cutaneous T-cell lymphoma (CTCL) such as Sezary Syndrome (SS) or erythrodermic mycosis fungoides are not the most common diagnoses in such cases, but establishing a proper diagnosis plays a pivotal role in disease prognosis, treatment option, and patients survival. In many cases, a definite diagnosis based on histopathology and immunohistochemistry may be difficult and clinicopathological correlation is essential for immunostaining interpretation^1^.

Nowadays dermoscopy is widely applied not only for preoperative evaluation of pigmented skin tumors, but also for alopecia diagnosis and is named trichoscopy^2,3^, for nail disorders—onychoscopy^4,5^ and inflammatory skin diseases with a name inflammoscopy^6–8^.

There are only few papers regarding dermoscopy of mycosis fungoides (MF). Lallas et al.^9^ evaluated 32 patients diagnosed with early patch stage of MF. They described fine, short, linear vessels, spermatozoa-like vascular structures and orange-yellow patchy areas. Bosseila et al.^10^ after analysis of 25 patients diagnosed with MF described dotted vessels as the most common vascular pattern of MF skin lesions, followed by linear pattern.

Dermoscopy of poikilodermatous MF has been first described by Xu and Tan^11^. It was case report of a 59-year old patient. Dermoscopic pattern consisted of numerous polygonal structures containing white lobules divided by septa of pigmented dots and studded with fine red dots or hairpin vessels.

Ghahrmani et al.^12^ evaluated seven patients diagnosed with patch and plaque MF. They described the presence of regular-appearing interconnected white structureless patches encircling small fine linear vessels (fenestrated to trabeculated pattern of patchy white structureless areas). This feature was also present in 60% of patients diagnosed with folliculotropic mycosis fungoides. Additional findings included follicular erosions surrounded by dotted (40%) and fine linear vessels (40%), loss of terminal follicles, and comedo-like openings (60%).

### Objective

Assuming that hair follicles can be involved in erythrodermic CTCL and folliculotropism of atypical lymphocytes should affect hair epithelium differentiation and maturation resulting in hair shafts abnormalities, the study regarding the value of scalp dermoscopy (trichoscopy) in differentiation between erythrodermic CTCL (erythrodermic MF or Sezary Syndrome), erythrodermic psoriasis, and erythrodermic atopic dermatitis was designed. To the best of our knowledge there is no study regarding trichoscopy in erythrodermic CTCL already published.

### Material and methods

The analysis included 16 patients diagnosed with erythrodermic MF or SS. All of them had confirmed diagnosis based on histopathology, immunohistochemistry and in case of SS detection of monoclonal atypical circulating CD4+ lymphocytes in increased number predominating ten times over CD8+ cells^13^. Clonality of atypical T CD4+ was confirmed by PCR (TCR rearrangement). As a comparative group 20 patients with psoriatic erythroderma and 20 patients diagnosed with erythrodermic atopic dermatitis were stated. All patients were examined at our department between July 2016 and March 2020. As the control, 20 healthy volunteers age and sex matched to investigated groups, were examined by trichoscopy.

In each patient demographic and clinical information including sex and comorbidities was collected. In each patient, trichoscopy was performed with the use of Fotofinder digital dermoscope (at a 20- and 70-fold magnification); 10–30 pictures were taken and analyzed for each examination.

All trichoscopic images (1119 images in total) were analyzed by two independent blinded evaluators (experts in dermoscopy) for the presence of hair shaft structure abnormalities, skin surface appearance, type and arrangement of blood vessels. After evaluation, results were unblinded and compatible dermoscopic features were assigned to respective patients groups (compatibility between evaluators: 98%).

The statistical analysis of the data was conducted using R GNU software. The differences in the incidence rate of various trichoscopic features amongst patients with erythrodermic CTCL, erythrodermic psoriasis, erythrodermic atopic dermatitis, and control group were examined with a chi-squared test with alpha correction for multiple comparisons The results were considered statistically significant with *p*-values lower than 0.05. Sensitivity, specificity, positive and negative prognostic value, and diagnostic odds ratio were calculated for each trichoscopic feature.

The study protocol was approved by the Medical University of Warsaw Review Board for Ethics in Human Research (protocol number AWBE/90/16). All experiments were performed in accordance with relevant named guidelines and regulations. Informed consent was obtained from all participants and/or their legal guardian/s.

## Results

The analysis included 16 patients with erythrodermic T-cell lymphoma [ten men and six women; mean age 56 (range 46–71)], 20 with psoriatic erythroderma [12 men, eight women; mean age 51 (range 38–67)], 20 patients diagnosed with erythrodermic atopic dermatitis [11 men and nine women; mean age 31 (range 19–51)] and 20 healthy volunteers [six men, 14 women; mean age 41 (range 26–54)].

The observed trichoscopic features included: numerous *pili torti*, singular *pili torti*, numerous broken hairs, singular broken hair, eight-shaped hairs (newly described thin, short, pigmented hair shafts, repeatedly rolled up in opposites sites), black dots, yellow dots, linear perifollicular vessels, glomerular perifollicular vessels, glomerular vessels regularly distributed or clustered, dotted vessels, arborizing vessels, glomerular vessels arranged in lines or circles localized between follicular units, white thick interfollicular bands forming net, patchy hyperpigmentation, yellow interfollicular scaling, white interfollicular scaling, follicular spicules like scaling.

**Figure 1:**
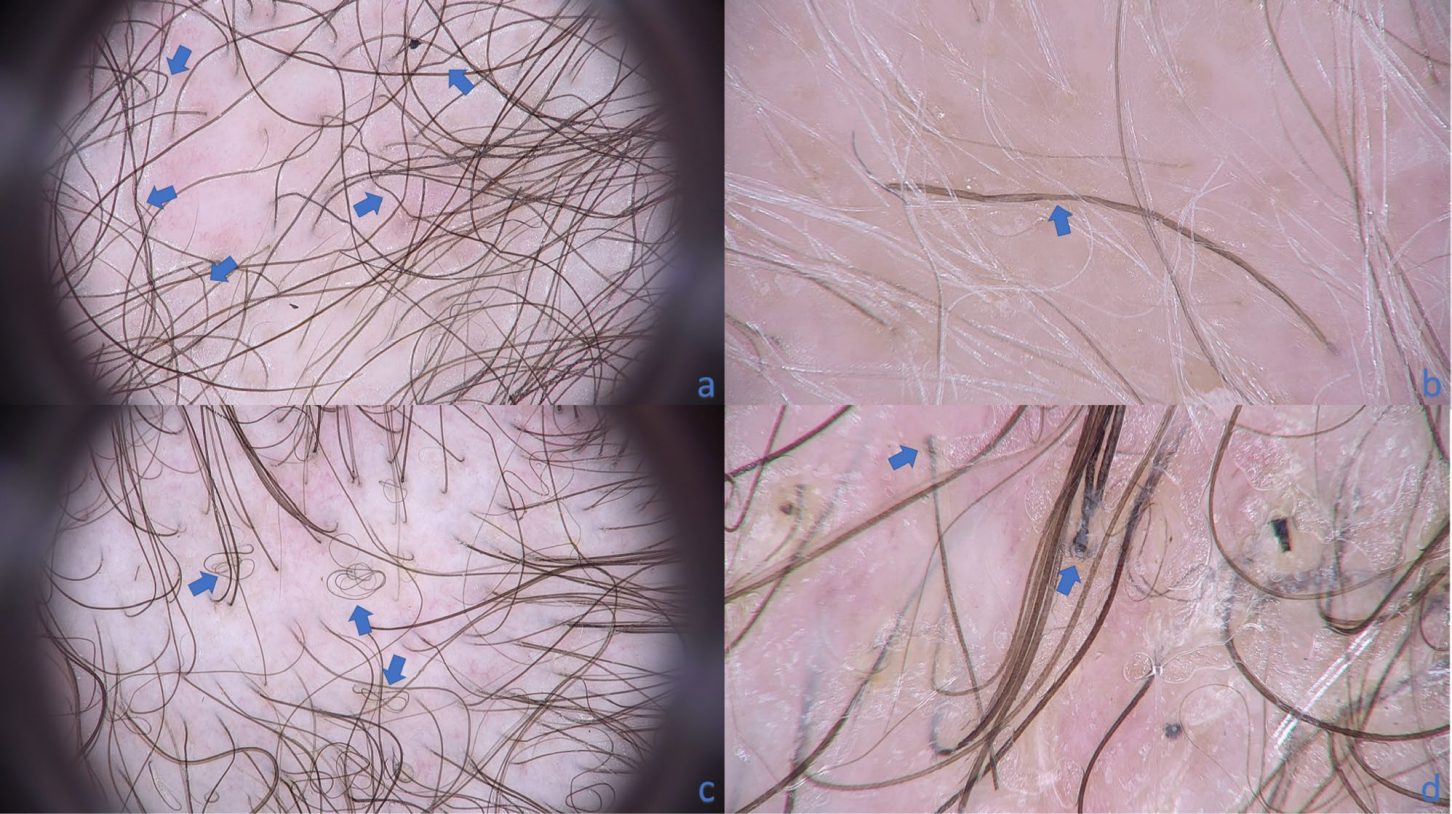
Hair shafts abnormalities seen in patient with erythrodermic CTCL. **(a)** Numerous *pili torti* seen in trichoscopy as bent hair shafts (blue arrow) (× 20). **(b)** Trichoscopy without immersion fluid and in high magnification shows short hair shaft with torsions along own axis (marked with blue arrow) (× 70).** (c)** Trichoscopy of erythrodermic CTCL with numerous hair shafts described as 8-shaped hairs (blue arrows) (× 20). **(d)** Visible anagen bulbs (blue arrow) (× 70).

Numerous *pili torti* (Fig. 1a,b), numerous broken hairs, white thick interfollicular bands forming net and patchy hyperpigmentation of the background were the most common and pathognomonic features for erythrodermic CTCL. They were observed in 81% (13/16), 75% (12/16), 56% (9/16) and 37.5% (6/16) of patients with cutaneous lymphomas, respectively (Table 1). They were not present in patients with erythrodermic psoriasis, erythrodermic atopic dermatitis and healthy volunteers (p < 0.001).

**Table 1:** Descriptive statistics among specific groups of patients: erythrodermic atopic dermatitis, control group, erythrodermic psoriasis, erythrodermic variants of cutaneous T-cell lymphoma (CTCL).

	Atopic dermatitis (N = 20)	Control group (N = 20)	Psoriasis (N = 20)	CTCL (N = 16)	p-value
**Hair shaft**					
Numerous* pili torti*	0% (0/20)	0% (0/20)	0% (0/20)	81% (13/16)	< 0.001
Visible anagen bulbs	0% (0/20)	0% (0/20)	0% (0/20)	13% (2/16)	0.053
8-shaped hairs	0% (0/20)	0% (0/20)	0% (0/20)	19% (3/16)	< 0.05
Solitary *pili torti*	0% (0/20)	0% (0/20)	0% (0/20)	13% (2/16)	0.053
Numerous broken hairs	0% (0/20)	0% (0/20)	0% (0/20)	75% (12/16)	< 0.001
Solitary broken hairs	25% (5/20)	0% (0/20)	10% (2/20)	19% (3/16)	0.107
**Follicular ostia**					
Yellow dots	50% (10/20)	30% (6/20)	15% (0/20)	88% (14/16)	< 0.05
Black dots	0% (0/20)	0% (0/20)	0% (0/20)	25% (4/16)	< 0.01
**Blood vessels**					
Linear perifollicular vessels	5% (1/20)	0% (0/20)	0% (0/20)	31% (5/16)	< 0.01
Glomerular perifollicular vessels	10% (2/20)	0% (0/20)	0% (0/20)	50% (8/16)	< 0.001
Glomerular regularly distributed vessels	20% (4/20)	5% (1/20)	100% (20/20)	50% (8/16)	< 0.01
Dotted vessels	95% (19/20)	75% (15/20)	10% (2/20)	25% (4/16)	< 0.01
Arborizing vessels	20% (4/20)	50% (10/20)	0% (0/20)	31% (5/16)	< 0.01
Glomerular line arranged vessels	0% (0/20)	0% (0/20)	100% (20/20)	13% (2/16)	< 0.001
**Background**					
White thick interfollicular bands	0% (0/20)	0% (0/20)	0% (0/20)	56% (9/16)	< 0.001
Patchy hyperpigmentation	0% (0/20)	0% (0/20)	0% (0/20)	38% (6/16)	< 0.001
Reddish areas	15% (3/20)	5% (1/20)	10% (2/20)	75% (12/16)	< 0.001
**Scale**					
Yellow interfollicular scales	95% (19/20)	0% (0/20)	10% (2/20)	25% (4/16)	< 0.001
White interfollicular scales	15% (3/20)	20% (4/20)	90% (18/20)	88% (14/16)	< 0.01
Follicular spicules-like scales	0% (0/20)	0% (0/20)	0% (0/20)	13% (2/16)	0.053

Among those features only two do not perform any statistically significant differences for inter-group comparisons: visible anagen bulbs (p = 0.053) and single broken hairs (p > 0.1). For the other features further multiple comparison test was applied.

In two of three patients diagnosed with erythrodermic CTCL and without numerous *pili torti*, singular *pili torti* were seen and they were not observed in other study groups.

Other specific features for erythrodermic CTCL were 8-shaped hairs (Fig. 1c) and visible anagen bulbs (Fig. 1d). Although they were observed in 19% (3/16) and 12.5% (2/16) of patients, they were not present in other patients groups (p < 0.05 and p = 0.052, respectively).

Singular broken hairs were seen in 25% (5/20) of patients with atopic dermatitis and 10% (2/20) of patients with erythrodermic psoriasis, but their presence was not statistically significant (p > 0.1).

Black dots were found in 25% (4/16) of patients with cutaneous lymphoma and were not seen in other study groups (p < 0.05).

Yellow dots were observed in all patients groups; they were present in 87% (14/16) of erythrodermic CTCL, 19% (3/20) of psoriasis group, 50% (10/20) of atopic dermatitis group and 30% (6/20) of the control group. Their presence in general excludes cicatricial alopecia.

Large reddish areas were observed in 75% (12/16; p < 0.001) of patients with erythrodermic CTCL. Although they were also observed in 10% (2/20) of patients with erythrodermic atopic dermatitis and erythrodermic psoriasis, and in 5% (1/20) of the control group.

**Figure 2:**
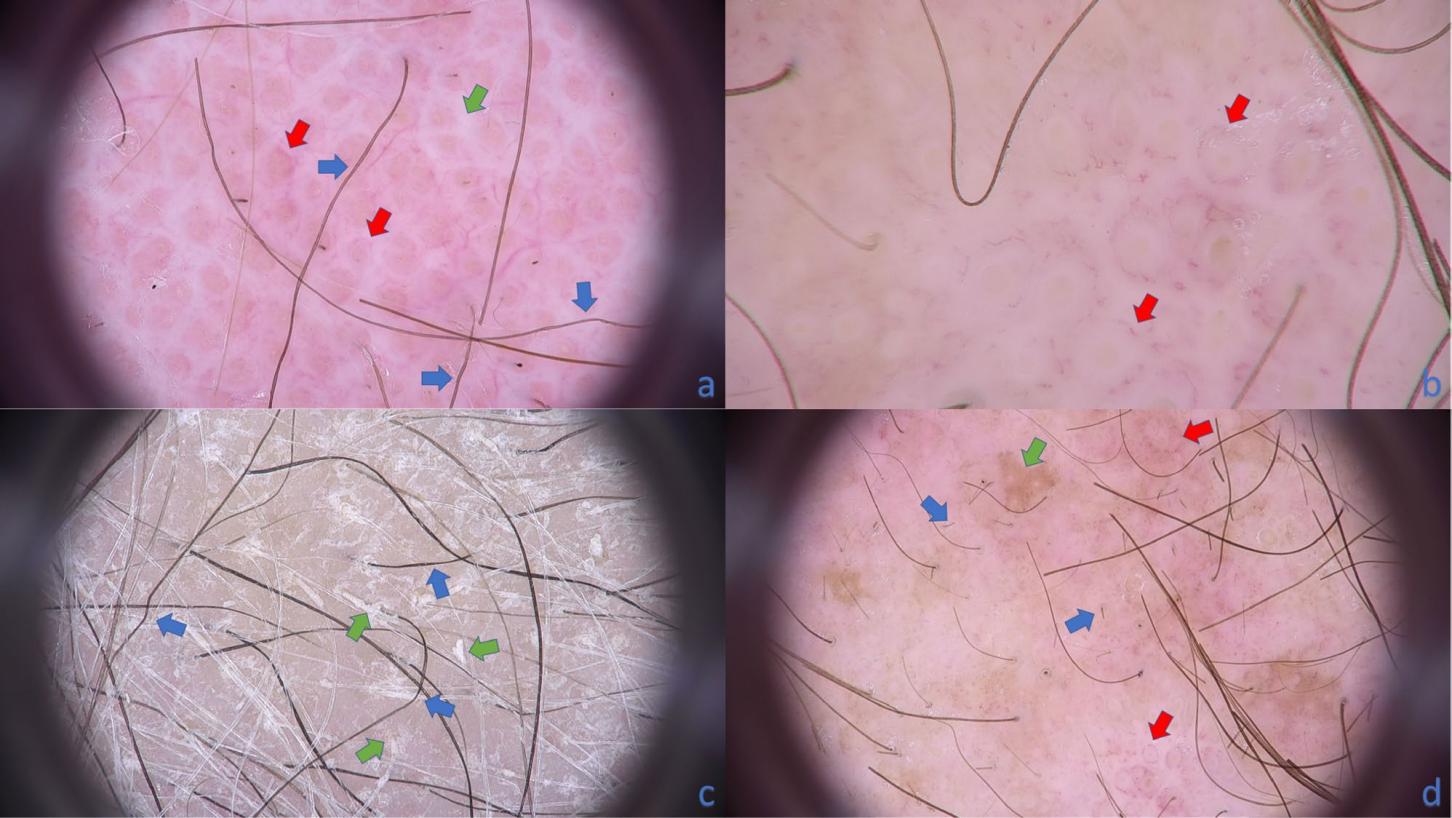
Trichoscopy of patient with erythrodermic CTCL. **(a)** The characteristic trichoscopic features visible on this image are: *pili torti* (blue arrows), thick white bands localized interfollicularly (green arrow) and perifollicular arrangement of linear vessels (red arrows)(× 20). **(b)** Vessels perifollicular arrangement can be easily recognized (red arrows). Short linear vessels are seen around yellow dots (empty follicular openings field with keratin mass and sebum)(× 70). **(c)** Numerous *pili torti* are marked with blue arrows. Additional feature seen if trichoscopy is performed without immersion fluid is follicular spicules-like scaling (green arrows)(× 20). **(d)** Short broken hairs are marked with blue arrows, perifollicular vessels arrangement with red arrows and patchy brownish discoloration of background with green arrow (× 20).

The most common vascular pattern of erythrodermic CTCL patients was perifollicular arrangement of glomerular (50%; 8/16; p < 0.001) or linear vessels (31%; 5/16; p < 0.05) (Fig. 2a,b). However, it was observed also in atopic dermatitis (10%; 2/20 and 5%; 1/20, respectively).

Follicular spicules-like scaling (Fig. 2c) was rare but pathognomonic for erythrodermic CTCL (12%, 2/16) although its presence did not reach statistical significance (p = 0.052).

Sensitivity, specificity, positive and negative likelihood ratio and diagnostic odd ratio of trichoscopic criteria for erythrodermic CTCL, erythrodermic atopic dermatitis and erythrodermic psoriasis are presented in Tables 2, 3 and 4.

**Table 2:** Sensitivity, specificity, positive and negative likelihood ratio and diagnostic odd ratio of trichoscopic criteria for erythrodermic cutaneous T-cell lymphoma vs erythrodermic psoriasis and erythrodermic atopic dermatitis.

Trichoscopic features	Sensitivity (%)	Specificity (%)	Positive likelihood ratio	Negative likelihood ratio	Diagnostic odds ratio
Numerous *pili torti*	81	93	11.65	0.20	57.8
Visible anagen bulbs	13	74	0.48	1.18	0.4
8-shaped hairs	19	75	0.76	1.08	0.7
Singular* pili torti*	13	74	0.48	1.18	0.4
Numerous broken hairs	75	91	8.25	0.28	30.0
Singular broken hairs	19	72	0.66	1.13	0.6
Numerous yellow dots	88	93	12.69	0.13	94.5
Black dots	25	77	1.08	0.98	1.1
Linear perifollicular vessels	31	78	1.42	0.88	1.6
Glomerular perifollicular vessels	50	83	2.88	0.61	4.8
Glomerular regularly distributed vessels	50	67	1.50	0.75	2.0
Dotted vessels	25	61	0.65	1.22	0.5
Arborizing vessels	31	77	1.34	0.90	1.5
Glomerular line arranged vessels	13	59	0.30	1.49	0.2
White thick interfollicular bands	56	85	3.78	0.51	7.3
Patchy hyperpigmentation	38	80	1.88	0.78	2.4
Reddish areas	75	90	7.31	0.28	26.3
Yellow interfollicular scales	25	61	0.65	1.22	0.5
White interfollicular scales	88	90	9.19	0.14	66.5
Follicular spicules-like scales	13	74	0.48	1.18	0.4

**Table 3:** Sensitivity, specificity, positive and negative likelihood ratio and diagnostic odd ratio of trichoscopic criteria for erythrodermic atopic dermatitis vs erythrodermic psoriasis and erythrodermic cutaneous T-cell lymphoma.

Trichoscopic features	Sensitivity (%)	Specificity (%)	Positive likelihood ratio	Negative likelihood ratio	Diagnostic odds ratio
Numerous *pili torti*	0	64	0.00	1.57	0.00
Visible anagen bulbs	0	94	0.00	1.06	0.00
8-shaped hairs	0	92	0.00	1.09	0.00
Singular *pili torti*	0	94	0.00	1.06	0.00
Numerous broken hairs	0	67	0.00	1.50	0.00
Singular broken hairs	25	86	1.80	0.87	2.07
Numerous yellow dots	50	53	1.06	0.95	1.12
Black dots	0	89	0.00	1.13	0.00
Linear perifollicular vessels	5	86	0.36	1.10	0.33
Glomerular perifollicular vessels	10	78	0.45	1.16	0.39
Glomerular regularly distributed vessels	20	22	0.26	3.60	0.07
Dotted vessels	95	83	5.70	0.06	95.00
Arborizing vessels	20	86	1.44	0.93	1.55
Glomerular line arranged vessels	0	39	0.00	2.57	0.00
White thick interfollicular bands	0	75	0.00	1.33	0.00
Patchy hyperpigmentation	0	83	0.00	1.20	0.00
Reddish areas	15	61	0.39	1.39	0.28
Yellow interfollicular scales	95	83	5.70	0.06	95.0
White interfollicular scales	15	11	0.17	7.65	0.02
Follicular spicules-like scales	0	94	0.00	1.06	0.00

**Table 4:** Sensitivity, specificity, positive and negative likelihood ratio and diagnostic odd ratio of trichoscopic criteria for erythrodermic psoriasis vs erythrodermic atopic dermatitis and erythrodermic cutaneous T-cell lymphoma.

Trichoscopic features	Sensitivity (%)	Specificity (%)	Positive likelihood ratio	Negative likelihood ratio	Diagnostic odds ratio
Numerous* pili torti*	0	64	0.00	1.57	0.00
Visible anagen bulbs	0	94	0.00	1.06	0.00
8-shaped hairs	0	92	0.00	1.09	0.00
Singular *pili torti*	0	94	0.00	1.06	0.00
Numerous broken hairs	0	67	0.00	1.50	0.00
Singular broken hairs	10	78	0.45	1.16	0.39
Numerous yellow dots	15	33	0.23	2.55	0.09
Black dots	0	89	0.00	1.13	0.00
Linear perifollicular vessels	0	83	0.00	1.20	0.00
Glomerular perifollicular vessels	0	72	0.00	1.38	0.00
Glomerular regularly distributed vessels	100	67	3.00	0.00	NA
Dotted vessels	10	36	0.16	2.47	0.06
Arborizing vessels	0	75	0.00	1.33	0.00
Glomerular line arranged vessels	100	94	18.0	0.00	NA
White thick interfollicular bands	0	75	0.00	1.33	0.00
Patchy hyperpigmentation	0	83	0.00	1.20	0.00
Reddish areas	10	58	0.24	1.54	0.16
Yellow interfollicular scales	10	36	0.16	2.49	0.06
White interfollicular scales	90	53	1.91	0.19	10.06
Follicular spicules-like scales	0	94	0.00	1.06	0.00

Description of trichoscopic results seen in erythrodermic CTCL with its possible histopathological correlation is presented in details in Table 5.

**Table 5:** Description of trichoscopic findings in erythrodermic cutaneous T-cell lymphoma (CTCL) with histopathological correlation.

Trichoscopic features	Description	Possible histopathological correlation
*Pili torti*	Twisted dystrophic hair shafts	Folliculotropic inflammation without or with mucinous degeneration of the hair follicle induces pressure in the epithelium affecting hair shaft formation
Broken hairs	Short hairs with irregular distal ends; in CTCL they are the result of the breakage of *pili torti*	Folliculotropic inflammation without or with mucinous degeneration of the hair follicle induces pressure in the epithelium and influences hair shaft strength
Black dots	Residues of pigmented hairs broken or destroyed at scalp level; in CTCL they are the result of the breakage of *pili torti*	Folliculotropic inflammation without or with mucinous degeneration of the hair follicle induce pressure in the epithelium and influences hair shaft strength
Numerous yellow dots	Empty follicular openings filled with sebum and keratin	Indication of non-cicatricial alopecia caused by folliculotropic inflammation suppresing hair epithelium proper function
8-shaped hairs	Long, pigmented, very thin hairs which are repeatedly rolled in two opposite directions forming structure resembling number 8	Folliculotropic inflammation without or with mucinous degeneration of the hair follicle induces pressure in the epithelium and results in hair thinning
Visible anagen bulbs	Rectangular and dark pigmented hair bulbs, which are seen on scalp surface	Can be the result of elevation of the root of the anagen bulb induced by expanding upwards folliculotropic infiltration
Big, reddish areas	Red, patchy background color sized of a few follicular units	Intra- and perifollicular inflammatory infiltrate from the bulb to the infundibulum
Patchy hyperpigmentation of the background	Brownish patchy background color sized of a few follicular units	Lichenoid infiltrate of the epidermis or upper part of hair epithelia with keratinocytes damage and pigment release
Thick white bands localized interfollicularly	Whitish background color seen as thick bands between follicular units	They correspond probably to the pushed aside and upwards collagen fibers by surrounding inflammatory infiltrate, mucin deposits and hair epithelium degeneration
Perifollicular arrangement of glomerular or linear vessels	Glomerular or linear vessels localized around follicular units	Heavy perifollicular lymphocytic infiltrate pushes up and flatten superficial vascular plexus to form glomerular and linear perifollicular arrangement
Follicular spicules-like scales	Triangular in shape keratotic structures emerging from follicular orifices	Perifollicular inflammation and lymphocyte epidermotropism affect keratinocyte maturation and desquamation leading to spicules formation

## Discussion

To the best of our knowledge there are limited studies regarding trichoscopy in patients with mycosis fungoides. Four cases have been described, in which two patients were diagnosed with erythrodermic mycosis fungoides. There is no paper regarding dermoscopy or trichoscopy in Sezary Syndrome.

Slawinska et al.^14^ described trichoscopy of a 88-year old female patient diagnosed with MF who presented patchy alopecia. The authors observed decreased number of pilosebaceous units, mostly single hairs, milky-white globules, yellow dots with or without centrally located black dots/broken hairs, short hairs with split end, short broken hair, pigtail appearance hair, areas where follicles were replaced by white dots and lines, white and yellow scale. Second case report of folliculotropic MF in the aspect of trichoscopy was described by Souissi et al. The authors presented the patient diagnosed with a rare variant of folliculotropic mycosis fungoides (FMF), named spiky FMF. The trichoscopic findings in this case were thick coats of keratinaceous debris around dilated openings and hair shafts^15^. Miteva et al.^16^ described two patients with alopecia universalis associated with erythrodermic mycosis fungoides. Trichoscopic features described by authors were diffuse erythema, empty follicular openings (some contained hairs) filled in with keratotic plugs or filiform spicules, black dots, and sparse broken hairs.

In presented study the majority of pathognomonic features for erythrodermic CTCL arrived from specific hair shafts changes. The most sensitive and specific features for erythrodermic CTCL were numerous *pili torti* (sensitivity 81%; specificity 93%, positive likelihood ratio 11.65, negative likelihood ratio 0.20, diagnostic odds ratio 57.8; p < 0.001) (Fig. 1a,b).

By definition of *pili torti*, sections of a hair shaft are flattened at irregular intervals and rotated 180° around its long axis, with each twist being 0.4 to 0.9 mm in width^17^. *Pili torti* may be either inherited or acquired. The most common congenital defects and syndromes associated with pili torti include: Ronchese syndrome, Beare syndrome, Bjornstad syndrome, Menkes syndrome, Rapp-Hodgkin syndrome, trichodysplasia-xeroderma, trichothiodystrophy among other rarer inherited diseases and syndromes^18–20^. Acquired forms of *pili torti* may result from repetitive trauma, oral retinoid treatment, anorexia, inflammation in cicatricial alopecia, graft-vs-host disease, scleroderma and after hair transplantation^21,22^. In acquired *pili torti*, it is presumed that uneven perifollicular fibrosis causes rotational forces on the inner root sheath that distort the hair follicle inducing formation of *pili torti* that may easily break, leaving broken hairs or black dots^17^. Another, more convincing explanation of *pili torti* formation is unequal developing of the outer root sheath cells, leading to an irregular thickness of the outer root sheath and hair shaft formation with the subsequent twisting seen on trichoscopy^20^.

**Figure 3:**
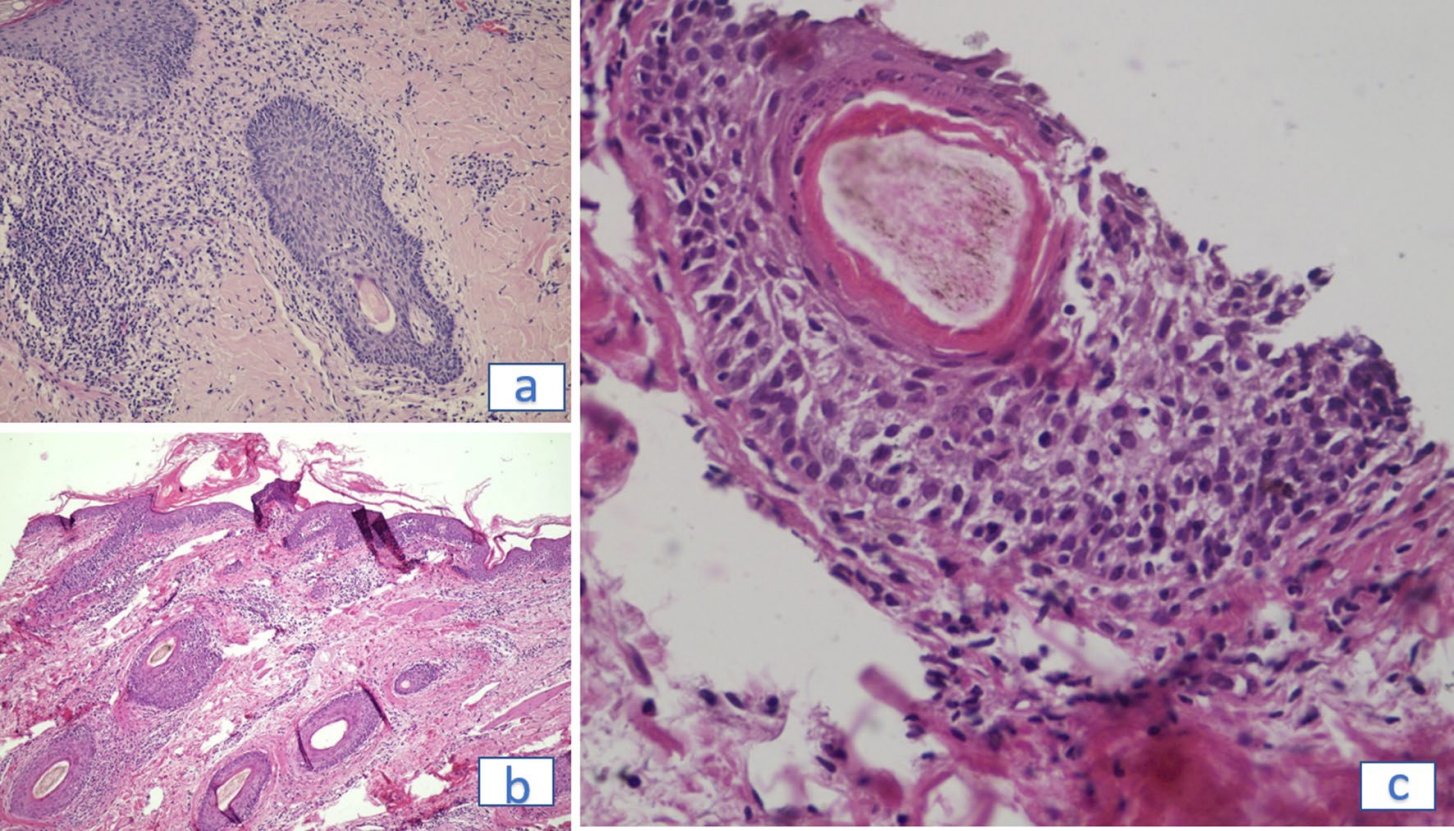
Scalp histopathology in erythrodermic CTCL. **(a)** Hair follicles with folliculotropism of atypical lymphocytes and uneven thickness of the outer root sheath (H&E, x200). **(b)** On the epidermal surface the prominent follicular keratotic spicule and interfollicular scale are visible in the scalp biopsy from the patient with erythrodermic mycosis fungoides. (H&E, × 100). **(c)** The fragment of a middle portion of the pili torti obtained in trichoscopy guided biopsy. The oval smooth shape of the hair shaft is lost. The hair epithelium is changed by lymphocytic inflammation, which leads to irregular thickness of the outer root sheath and the subsequent hair twisting. (H&E, × 400).

Acquired *pili torti* usually are observed in cicatricial alopecia such as lichen planopilaris, being the indication of the active lichenoid inflammatory infiltrate around upper part of the hair epithelium leading to thinning of the epithelium and induction of concentric lamellar fibrosis. Inflamed, distorted hair epithelium clenched by uneven concentric fibroplasia presents disturbed keratinization of the outer root sheath leading to the formation of the twisted hair shaft^20,23^. In the presented study acquired *pili torti* were found in majority of erythrodermic CTCL patients and were not observed in other types of erythroderma, such as psoriatic and atopic dermatitis. Their presence was not connected with the scarring process, which was excluded by the presence of yellow dots (empty follicular openings filled in with sebum and keratin). It can be hypothesized that folliculotropic inflammation without or with mucinous degeneration of the hair follicle, may induce pressure in the epithelium and *pili torti* formation (Fig. 3a,b,c). Therefore presence of numerous *pili torti* in trichoscopy of erythrodermic patient can be assumed as pathognomonic feature for erythrodermic mycosis fungoides or Sezary Syndrome, raising oncological vigilance.

Slawinska et al.^14^ described the presence of pigtail appearance hair, however they do not fit the classic description of this feature in the literature. The term circle hairs is used when hair has the same caliber along the hair shaft and is able to form a complete circle. Pig tail hair is thicker at the ostium, and it becomes more and more thin towards its end. Pig tail hair is assumed as the expression of thin regrowing anagen hair^24^. The eight-shaped hairs described in our study (Fig. 1c) are long pigmented very thin hair which are repeatedly rolled in two opposite directions forming structure resembling number 8. They were found only in patients with erythrodermic CTCL in 19% (3/16) and can be assumed as pathognomonic for this condition if they are numerous (sensitivity is 13% and specificity 75%; p < 0.05). Their appearance can be also explained by the presence of folliculotropic lymphocytic infiltration.

Visible anagen bulbs were previously described in aplasia cutis congenita^25^, lichen planopilaris^26^, and erosive pustular dermatosis of the scalp^27^. These all conditions belong to cicatricial alopecia group. In our study visible anagen bulbs were found only in erythrodermic CTCL (13%; 2/16), although their presence has not met statistical significance (p = 0.052). Visible anagen bulb in CTCL can be the result of elevation of the root of the anagen bulb induced by expanding upwards perifollicular and folliculotropic infiltration (Fig. 1d).

Broken hairs and black dots are the result of the breakage of *pili torti*. In our study numerous broken hairs were found in 75% (12/16) of CTCL patients and were not observed in other groups (p < 0.001) (Fig. 2d).

The background color seen in trichoscopy of erythrodermic CTCL patients was heterogenous. Big, reddish areas were observed in 75% (12/16) of those patients although they were also present in 15% of patients with erythrodermic atopic dermatitis and in one healthy individual. Patchy brownish background discoloration was observed only in patients with erythrodermic CTCL (38%; 6/16; p < 0.001) (Fig. 2d).

In 56% (9/16) of CTLC patients trichoscopy revealed thick white bands localized interfollicularly (Fig. 2a). They correspond most probably to the pushed aside and upwards collagen fibers induced by inflammatory infiltrate, mucin deposits, and degeneration of hair epithelium. This feature was not observed in other examined groups in presented study. Thick white bands may be trichoscopic equivalent of regularly-appearing interconnected white structureless patches encircling small fine linear vessels found in dermoscopy of MF in study of Ghrahramani et al.^12^. The difference may be derived by arrangement of hair follicles on glabrous skin (small vellus follicles which are densely placed) and scalp skin (bigger follicles producing terminal hair shafts which are localized in follicular units).

In previous reports vascular pattern found in patients with mycosis fungoides were fine, short, linear vessels, spermatozoa-like vascular structures and dotted vessels^9–12^.

Our study shows that in scalp examination of erythrodermic CTCL most pathognomonic pattern is perifollicular arrangement of glomerular or linear vessels (Fig. 2a,b) although it was not observed in 5 out of 16 patients and was present in 3 out of 20 patients diagnosed with erythrodermic atopic dermatitis. To the best of our knowledge it is first description of this particular vascular pattern.

The limitation of the study is small number of patients included into analysis and further investigations are needed.

## Conclusions

If numerous* pili torti* are found in scalp dermoscopy of patient with erythroderma, CTCL should be suspected.

The other dermoscopic features found in this cases are: eight-shaped hairs, thick white interfollicular bands, color heterogeneity of the background (large reddish areas, patchy brownish hyperpigmentation) and particular perifollicular arrangement of glomerular or linear vessels.
